# Examination of the action of the iliocapsularis: Focusing on changes in shear elastic modulus owing to muscle elongation

**DOI:** 10.1371/journal.pone.0325134

**Published:** 2025-06-04

**Authors:** Hikari Itsuda, Masahide Yagi, Hiroshige Tateuchi, Momoka Tokoro, Noriaki Ichihashi

**Affiliations:** 1 Human Health Sciences, Graduate School of Medicine, Kyoto University, Kyoto, Japan; 2 Research Fellow of Japan Society for the Promotion of Science, Chiyoda-ku, Tokyo, Japan; 3 Faculty of Rehabilitation, Kansai Medical University, Hirakata, Osaka, Japan; Nishikyushu University: Nishikyushu Daigaku, JAPAN

## Abstract

The iliocapsularis (IC) muscle contributes to hip stability and understanding its function *in vivo* is important. A previous study investigated the functional role of the IC by using electromyography. However, muscle can activate during not only their primary action, which is the direction to the moment arm, but also the stabilizers and fixators. As a result, clarifying the action of the muscle by measuring electromyography alone is a challenge. This study aimed to examine the action, namely the direction of the moment arm, of the IC using shear wave elastography. In total, 23 young healthy individuals (8 men and 15 women) participated in the study. The shear elastic modulus (*G*) of the right IC was measured in the following positions: (1) resting (hip flexion 0º, addiction 0º, and external rotation 0º), (2) hip flexion at 15º, (3) hip extension at 15º, (4) hip abduction at 15º, (5) hip adduction at 15º, (6) hip external rotation at 15º, and (7) hip internal rotation at 15º. Higher *G* values correspond to increased muscle elongation, assuming that the muscle acts in the opposite direction of the elevated *G*. Wilcoxon signed-rank test with Holm correction was performed to compare *G* between the resting position and each subsequent posture and between hip flexion–extension, hip abduction–adduction, and hip external–internal rotation. As a result, the *G* in extension (*p <* *0.001*) and external rotation (*p = 0.021*) were significantly higher than that in the resting position. *G* in extension was higher than in flexion (*p < 0.001*), and *G* in external rotation was higher than in internal rotation (*p = 0.007*). These findings suggest that the IC is involved in hip flexion and internal rotation.

## Introduction

The iliocapsularis (IC) muscle is attracting widespread interest in orthopedic research, particularly for its role in hip stability. Originating from the anterior inferior iliac spine (AIIS) and attaching to the anterior capsule and lesser trochanter [1–4], it may contribute to hip joint stabilization by maintaining the femoral head in an afferent position [5] and exerting tension on the joint capsule [4]. Moreover, the IC exhibits hypertrophy in patients with hip dysplasia, suggesting its potential role in compensating for bony instability [6,7]. Furthermore, IC activity has been reported to increase around toe-off during gait [8]. Therefore, the IC may be important for hip stability during sports with excessive hip motion, such as during ballet, gymnastics, and figure skating. Consequently, physical exercise therapy focusing on the IC may improve hip joint stability. Understanding muscle action is crucial for effective strength training and stretching. However, the specific action of the IC remains unclear, making it challenging to develop appropriate targeted interventions. Therefore, investigating the IC’s action is important.

Several studies have attempted to estimate the role of the IC by analyzing muscle activity. In one electromyographic study, the IC’s activities were measured during maximal voluntary isometric contraction of the hip. The study revealed that IC activity was most prominent during 90º hip flexion, followed by hip abduction, and then hip external rotation at 0º flexion [9]. Another electromyographic study with a focus on gait, reported that IC activity increased during mid to late stance in shortened strides compared to self-selected strides [8]. However, estimating muscle action solely from electromyography is challenging because muscles during voluntary contraction are activated not only as agonists but also as stabilizers and fixators [10–14]. In other words, clarifying the actions of the IC using electromyography is difficult.

Ultrasonic shear wave elastography (SWE) allows for the estimation of IC action [15]. However, although conventional methods using MRI have advantages for assessing muscle function, there are also several limitations to their use. First, muscle moment arm estimation using conventional MRI remains a challenge because it is difficult to accurately identify the IC’s line of action in three dimensions. This is due to conventional MRI only being able to visualize a portion of the IC. Second, while T2-weighted imaging effectively reflects metabolic or physiological stress on the muscle, it does not differentiate between active force generation and passive elongation [16], much less muscle contraction condition (agonist, stabilizers, and fixation). SWE may provide an alternative to overcome these problems. The shear elastic modulus (*G*) is computed through SWE, determined by the propagation velocity of shear waves generated by the acoustic radiation force upon irradiation of focused ultrasound pulses into the tissue [17]. A prior study established a high intra-individual correlation between the *G* values and the degree of muscle elongation that changes with joint angle; an elevated *G* value signifies increased muscle elongation [18]. This suggests that SWE can noninvasively assess the extent of muscle elongation *in vivo* [19,20]. Furthermore, the extent of muscle elongation impacts the joint angle in a direction opposite to that of the moment arm [21]. Therefore, if the joint angle change is constant, the direction of muscle action can be inferred to be opposite to the direction of muscle elongation, as measured by an increase in *G*. This method can overcome the limitations of electromyography in estimating muscle action because it does not require muscle contraction. A previous study measuring the *G* of the IC revealed that this muscle is substantially more stretched in external hip rotation than in the neutral hip position [22]. However, it is difficult to conclude IC’s action based on these results because the comparison was limited to the hip neutral position and external rotation. Furthermore, the external rotation angle was not defined.

In this study, the action of the IC using SWE, specifically the direction of the moment arm, was examined focusing on changes in *G* due to muscle elongation. Previous studies reported that the IC is more elongated in hip external rotation than in neutral position [23] and that its activity is greater in the directions of hip flexion and abduction [9]. Based on these findings, we hypothesized that the IC is elongated in hip extension, adduction, and external rotation. In other words, the IC has action of hip flexion, abduction, and internal rotation *in vivo*.

## Materials and methods

### Design

This study is an observational cross-sectional study.

### Participants

The participants were 23 healthy young individuals (men/women: 8/15, age 22.9 ± 1.3 years, height 162.0 ± 8.1 cm, body mass 52.9 ± 6.4 kg). The determination of the sample size was conducted using the Wilcoxon signed-rank test (matched pairs) model (effect size = 0.64 [24], α error = 0.05, Power = 0.8) in G*power 3.1 (Heinrich Hein University, Duesseldorf, Germany). The required sample size was determined to be 22 participants. The exclusion criteria were individuals with hip discomfort or pain, orthopedic disease in the lower extremity joints, or a history of previous orthopedic surgery. The participants were recruited during the period from October 1, 2021, to November 31, 2021. All participants were briefed in advance about the study, and provided written consent for their participation. The study protocol was approved by Kyoto University Graduate School and Faculty of Medicine, Ethics Committee (registration number: R0881).

### Procedure

**Fig 1:**
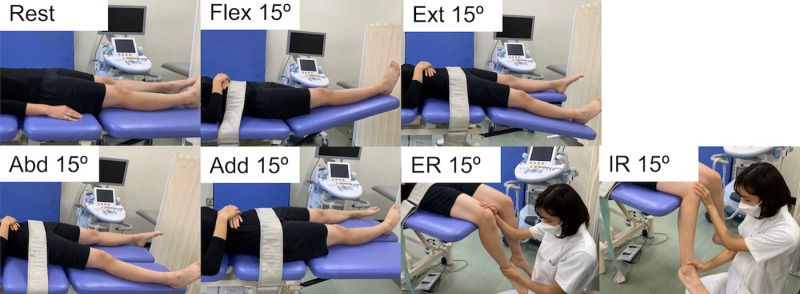
Measurement positions. Rest: hip flexion 0º hip abduction 0º hip external rotation 0º, Flex 15º: hip flexion 15º, Ext 15º: hip extension 15º, Abd 15º: hip abduction 15º, Add 15º: hip adduction 15º, ER 15º: hip external rotation 15º, IR 15º: hip internal rotation 15º.

The target muscle was the right IC. Participants were placed in a supine position, and measurements were taken in the following seven position (Fig 1); 1) Rest (hip flexion 0º, abduction 0º, external rotation 0º), 2) hip flexion 15º (Flex 15º), 3) hip extension 15º (Ext 15º), 4) hip abduction 15º (Abd 15º), 5) hip adduction 15º (Add 15º), 6) hip external rotation 15º (ER 15º), and 7) hip internal rotation 15º (IR 15º). Some participants could not be measured when the *G* exceeded hip flexion 15º of hip in preliminary experiments. Therefore, a hip angle of 15º was set for measuring *G* in all participants and positions. In Flex 15º and Ext 15º, only the right lower extremity underwent angulation in hip flexion and extension using a treatment table. In ER 15º and IR 15º, an examiner manually held the participant's lower limb in external or internal rotation. Furthermore, in all positions, the participants were instructed to relax, and their pelvis was secured to the bed using a belt to prevent pelvis compensations. Two consistent examiners conducted the measurements: one ensured pelvic and lower extremity fixation, whereas the other performed the ultrasound measurements.

### Measurement of the shear elastic modulus

**Fig 2:**
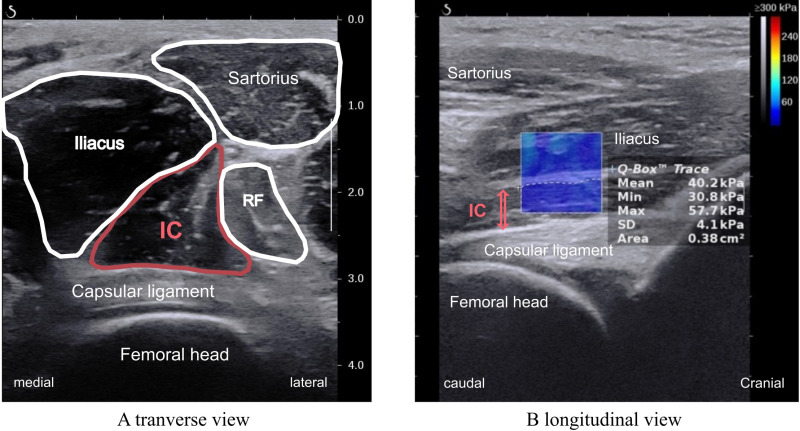
An example of the shear elastic modulus colormap image. IC: iliocapsularis, RF: rectus femoris.

The *G* was measured using SWE with an SL 10–2 linear probe (Aixplorer version 12.2.0, Supersonic Image, Aix-en-Provence, France). The ultrasonic settings were as follows: MSK–mode, penetration mode; frequency, 10-12 Hz (depending on the depth); persistence, medium; 100% opacity; 70% gain; and 5 smoothing. The *G* of the IC was measured at a point 4 cm distal to the anterior superior iliac spine [25]. Initially, the probe was placed in the short-axis direction of the femur to identify the IC belly (Fig 2A). Subsequently, the probe was then rotated to align parallel with the muscle fibers of the IC (Fig 2B), and the *G* color map was recorded while maintaining this probe position.

On the color map image, a 1-cm square region of interest (ROI) was set at the IC belly, carefully selected to represent the maximum area while excluding the fascia and capsule (Fig 2B). The SWE calculated the average *G* in the ROI based on *G* = *ρν*^2^, where *ρ* is the material density, and *ν* is the shear wave velocity. The density (*ρ)* of the muscle was assumed to be 1 g/cm^3^ [26]. The *G* exhibits a higher value when the muscle is more elongated [18], and the extent of muscle elongation is impacted by the moment arm, given a constant change in joint angle [27]. Therefore, in this study, muscle action was defined as the direction opposite to which the *G* was higher. This was determined by comparing the *G* values when moving the hip joint from rest to each direction, as well as between Flex 15º-Ext 15º, Abd 15º-Add 15º, and ER 15º-IR 15º.

Measurements were performed in a random order to eliminate order effects. Randomization was performed using the random function in Excel (Microsoft, Redmond, USA). The *G* at each position was measured three times, and the average value was used for the analysis. In addition, a 5-minute rest was taken between each position. In addition, *G* was measured within 30 seconds in each position, based on a previous study in which the *G* of the small muscle was found to decrease after 30 seconds of stretching [28].

SWE measurements were performed by a physical therapist with two years of experience in musculoskeletal ultrasound. The examiner was trained by a physical therapist with eight years of experience in musculoskeletal ultrasound, who published a previous study on ultrasound imaging of this specific muscle and supervised all measurements in that study [25].

### Statistical analysis

Intraclass correlation coefficients (ICC) (1,3) were calculated from three measurements at each position to examine the intrarater reliability of the mean *G*. The Shapiro–Wilk test was used to confirm the normality of *G*. Non-normality was observed in Ext 15º *(p = 0.034*) and IR 15º *(p = 0.017*). Therefore, the Wilcoxon signed-rank test with Holm correction was conducted to compare the *G* values between Rest and each position, Flex 15º-Ext 15º, Abd 15º-Add 15º, and ER 15º-IR 15º. The effect size *r* was estimated using the test statistics *Z* calculated by the Wilcoxon signed-rank test. The results are shown as adjusted p-values and *r.* Statistical significance was set at 5%. Statistical analyses were performed using SPSS, (version 29.0) (SPSS Japan, IBM SPSS Statistics, USA).

## Results

**Fig 3:**
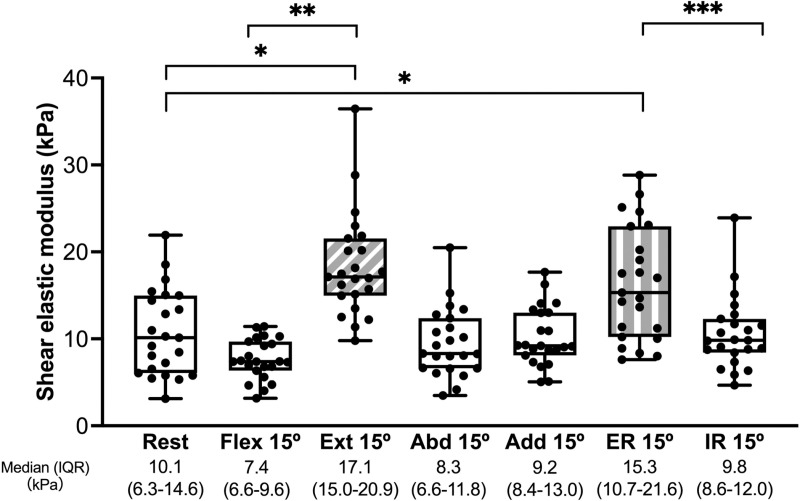
Shear elastic modulus of the IC in each position. Rest: hip flexion 0º, abduction 0º, external rotation 0º, Flex 15º: hip flexion 15º, Ext 15º: hip extension 15º, Abd 15º: hip abduction 15º, Add 15º: hip adduction 15º, ER 15º: hip external rotation 15º, IR 15º: hip internal rotation 15º, IQR: interquartile range. Data indicate the median (first and third quartiles). The bottom edge of the whiskers indicates the minimum value, top edge of the whiskers indicates the maximum value, the line in the middle of the box indicates the median value, the bottom edge of the box indicates the first quartile, and the top edge of the box indicates the third quartile. * Significant difference between the results of the Wilcoxon signed-rank test with Holm correction for Rest and other positions is shown ** Significant difference between Flex 15º and Ext 15º. *** Significant difference between ER 15º and IR 15º.

ICCs (1, 3) were >0.949 for all positions, indicating “excellent” measurement reliability [29] (Table 1). Fig 3 shows the *G* values of the IC at each position. The *G* value of the IC was significantly higher in Ext 15º (*p* < 0.001, *r* = 0.80) and ER 15º (*p* = 0.021, *r* = 0.61) than at rest. Additionally, *G* in Ext 15º was significantly higher than that in Flex 15º (*p* < 0.001, *r* = 0.88), and *G* in ER 15º was significantly higher than that in IR 15º (*p* = 0.007, *r* = 0.69) (Fig 3). In other comparisons, no significant differences were observed in *G* values (Fig 3). Although not statistically significant, *G* in Flex 15º tended to be lower than that at rest (*p* = 0.053, *r* = 0.53).

**Table 1:** ICCs (1,3) in each position.

Rest	Flex 15º	Ext 15º	Abd 15º	Add 15º	ER 15º	IR 15º
0.959 (0.920-0.981)	0.963 (0.927-0.983)	0.982 (0.965-0.992)	0.984 (0.974-0.993)	0.949 (0.900-0.976)	0.970 (0.941-0.986)	0.962 (0.926-0.982)

Rest: hip flexion 0º hip abduction 0º hip external rotation 0º, Flex 15º: hip flexion 15º, Ext 15º: hip extension 15º, Abd 15º: hip abduction 15º, Add 15º: hip adduction 15º, ER 15º: hip external rotation 15º, IR 15º: hip internal rotation 15º. Values indicate the average measures (95%CI).

## Discussion

This study investigated the action of the IC by elucidating the direction of hip joint motion in which the stiffness of IC increased using SWE. The findings revealed that the *G* of the IC was substantially higher in hip extension and external rotation compared to that at rest, the neutral hip position. Furthermore, the *G* of the IC was substantially higher in hip extension and external rotation compared to hip flexion and internal rotation, respectively. Specifically, the IC was confirmed to have actions of hip flexion and internal rotation, supporting our hypothesis. Nevertheless, there were no substantial findings for abduction–adduction, contradicting our initial hypothesis. To the best of our knowledge, this study is the first to evaluate the action of the IC, namely the direction of moment arm, using SWE in young healthy individuals. As a result, this study provides important insights into the kinematics of the IC.

The *in vivo* action of the IC was estimated using SWE. In a previous study, a high intraindividual correlation was reported between the degree of muscle elongation and *G*, with higher *G* values indicating greater muscle elongation [30]. Muscle action was estimated by examining *G* variations across joint directions, with the action inferred to be opposite the direction of increasing *G*, which reflects muscle elongation [15]. The results of the present study indicated that the IC showed a higher *G* at rest and during hip extension than during flexion; that is, the IC has an action in hip flexion. Similarly, a higher *G* in external rotation than in internal rotation suggests that the IC also plays a role in hip internal rotation.

IC was elongated in hip extension and external rotation, and tended to be loosened in hip flexion. These results were similar to those of a previous study where *G* of IC was substantially higher in hip external rotation than in neutral position [23]. However, because the absence of a prescribed angle for hip external rotation in the previous study and the lack of comparison with the *G* values in internal rotation, the extent to which our results can be directly compared with those findings is limited. Interestingly, anatomical studies have shown that the inferior iliofemoral ligament relaxes in hip flexion [31] and is elongated in hip extension and external rotation [32,33]. The inferior iliofemoral ligament originate from the anterior inferior iliac spine and insert at the lesser trochanter of the femur [1,2,34], suggesting that the IC and the ligament have similar pass and are located in the deep layer of the hip. Thus, it is plausible that the IC experienced elongation in hip extension and external rotation, displaying a tendency to loosen in hip flexion, similar to the inferior iliofemoral ligament.

We hypothesized that the IC has a hip abduction action besides hip flexion and internal rotation; however, our findings did not reveal any notable action in the coronal plane. These findings seem to contrast with a previous study that reported the increased IC activity during maximal isometric hip abduction [9]. Neumann et al. defined muscle action as the joint movement resulting from muscle contraction [35], implying that increased muscle activity can serve as an estimate of muscle action. However, it is crucial to note that muscles serve not only as agonists but also take on roles as stabilizers and fixators [10–14]. Additionally, considering that the IC runs longitudinally to the anterior femoral head [36], its impact on the abduction–adduction moment may be minimal because of its position near the center of hip joint in the transverse plane. Hence, the IC may not directly contribute to hip abduction. However, it may play an important role in activating during abduction and fixation of the hip joint, as other muscles [10–14]. Further research is required to explore this aspect.

These results indicate that the IC plays a role in hip flexion and internal rotation, which may have practical implications for optimizing training and rehabilitation strategies. Specifically, targeted stretching and strengthening exercises that emphasize IC action could benefit individuals recovering from hip disease or seeking to improve athletic performance. Furthermore, individual differences in *G* across hip joint positions may be influenced by hip joint laxity, with individuals exhibiting greater laxity showing less variation in *G* due to reduced passive stiffness [37]. These insights highlight the importance of considering muscle function and joint characteristics when designing personalized interventions for hip stability.

Our study had some limitations. First, the extent of joint angle change was as small as 15º for each direction of hip joint. The results might differ if larger joint angles were used. However, considering that the moment arm is estimated by changes in the joint angle and muscle length, the extent of muscle elongation had to be compared at a constant joint angle change. Nonetheless, we had to standardize the magnitude of joint angle, which could affect the *G*, because a comparison of *G* between different joint directions was required. Furthermore, if the flexion angle exceeds 15º, measuring the *G* of the IC using SWE becomes challenging. Therefore, 15º was chosen for this study. Second, our study included only healthy young adults. Therefore, the effects of bony morphology, such as variations in the femoral neck shaft and anteversion angles, on muscle action remain unclear. However, research in healthy participants was necessary to examine the original action of the muscle. Third, we were unable to measure IC activity during the *G* measurement to ensure the participants relaxation. However, measuring IC activity non-invasively is difficult because this muscle is located deep within the body. Fourth, the sample size may be insufficient to detect small differences between hip positions; therefore, we address only major actions of the IC. Fifth, *G* was measured at only one site. We may not have adequately assessed the overall *G* of the IC because the IC is a long muscle [4] and may not be elongated uniformly. Lastly, we did not examine sex differences in IC action because this was beyond the scope of this study, and the sample size after grouping by sex would have been insufficient. However, different results may be obtained between sexes when measuring SWE at larger joint angles or using a large sample size.

## Conclusion

The action of IC was investigated in healthy young participants based on change in *G* using SWE. This study found that the IC had the action of hip flexion and internal rotation. The findings of this study provide useful information for investigating the optimal physical exercise therapy of IC *in vivo*.
